# Retraction Note: Al Kharj diabetic patients’ perception about diabetes mellitus using revised-illness perception questionnaire (IPQ-R)

**DOI:** 10.1186/s12875-019-1043-3

**Published:** 2019-11-05

**Authors:** Sameer Al-Ghamdi, Gulfam Ahmad, Ali Hassan Ali, Nasraddin Bahakim, Salman Alomran, Waleed Alhowikan, Salman Almutairi, Tariq Basalem, Faisal Aljuaid

**Affiliations:** 1 Department of Family Medicine, College of Medicine, Prince Sattam bin Abdulaziz University, Al Kharj, Saudi Arabia; 2 Department of Basic Medical Sciences, College of Medicine, Prince Sattam bin Abdulaziz University, Al Kharj, Saudi Arabia; 3 Department of Anatomy, College of Medicine, Prince Sattam bin Abdulaziz University, Al Kharj, Saudi Arabia; 4 Department of Anatomy, College of Medicine, Alazhar University, Cairo, Egypt; 5 Department of Basic Medical Sciences, College of Medicine, Prince Sattam bin Abdulaziz University, Al Kharj, Saudi Arabia; 6 College of Medicine, Prince Sattam bin Abdulaziz University, Al Kharj, Saudi Arabia

**Retraction Note: BMC Fam Pract (2018) 19:21**

**https://doi.org/10.1186/s12875-018-0713-x**

The editors have retracted this article [1] because it contains extensive overlap with an article published by the same authors in the *Journal of Family & Community Medicine* [2] and is therefore redundant. The authors have not responded to correspondence from the editor about this retraction.

1. Al-Ghamdi S, Ahmad G, Ali AH, Bahakim N, Alomran S, Alhowikan W, et al. Al Kharj diabetic patients’ perception about diabetes mellitus using revised-illness perception questionnaire (IPQ-R). BMC Family Practice. 2018;19:21
2. Al-Ghamdi SH, Ahmad GAU, Ali AH, Bahakim NO, Alomran SI, Alhowikan WK, et al. How do Saudi diabetic patients perceive their illness? A multicenter survey using revised-illness perception questionnaire. Journal of Family & Community Medicine, 2018;25:2,75–8
